# The pathogenic role of succinate-SUCNR1: a critical function that induces renal fibrosis via M2 macrophage

**DOI:** 10.1186/s12964-024-01481-5

**Published:** 2024-01-30

**Authors:** Min Pu, Jing Zhang, Fuyan Hong, Yan Wang, Chengwei Zhang, Yongcheng Zeng, Zhenzhen Fang, Weiwei Qi, Xia Yang, Guoquan Gao, Ti Zhou

**Affiliations:** 1 Department of Biochemistry and Molecular Biology, Zhongshan School of Medicine, Sun Yat-sen University, Guangzhou, China; 2 Program of Molecular Medicine, Affiliated Guangzhou Women and Children’s Hospital, Zhongshan School of Medicine, Sun Yat-Sen University, Guangzhou, China; 3 Guangdong Engineering & Technology Research Center for Gene Manipulation and Biomacromolecular Products, Sun Yat-sen University, Guangzhou, China; 4 China Key Laboratory of Tropical Disease Control (Sun Yat-sen University), Ministry of Education, Guangzhou, China; 5 Guangdong Province Key Laboratory of Brain Function and Disease, Zhongshan School of Medicine, Sun Yat-Sen University, Guangzhou, China; 6 Guangdong Provincial Key Laboratory of Diabetology, Guangzhou, Guangdong China; 7 Department of Ultrasound, Chongqing Key Laboratory of Ultrasound, Molecular Imaging, The Second Affiliated Hospital of Chongqing Medical University, Chongqing, China

## Abstract

**Background:**

Renal fibrosis significantly contributes to the progressive loss of kidney function in chronic kidney disease (CKD), with alternatively activated M2 macrophages playing a crucial role in this progression. The serum succinate level is consistently elevated in individuals with diabetes and obesity, both of which are critical factors contributing to CKD. However, it remains unclear whether elevated succinate levels can mediate M2 polarization of macrophages and contribute to renal interstitial fibrosis.

**Methods:**

Male C57/BL6 mice were administered water supplemented with 4% succinate for 12 weeks to assess its impact on renal interstitial fibrosis. Additionally, the significance of macrophages was confirmed in vivo by using clodronate liposomes to deplete them. Furthermore, we employed RAW 264.7 and NRK-49F cells to investigate the underlying molecular mechanisms.

**Results:**

Succinate caused renal interstitial macrophage infiltration, activation of profibrotic M2 phenotype, upregulation of profibrotic factors, and interstitial fibrosis. Treatment of clodronate liposomes markedly depleted macrophages and prevented the succinate-induced increase in profibrotic factors and fibrosis. Mechanically, succinate promoted CTGF transcription via triggering SUCNR1-p-Akt/p-GSK3β/β-catenin signaling, which was inhibited by SUCNR1 siRNA. The knockdown of succinate receptor (SUCNR1) or pretreatment of anti-CTGF(connective tissue growth factor) antibody suppressed the stimulating effects of succinate on RAW 264.7 and NRK-49F cells.

**Conclusions:**

The causative effects of succinate on renal interstitial fibrosis were mediated by the activation of profibrotic M2 macrophages. Succinate-SUCNR1 played a role in activating p-Akt/p-GSK3β/β-catenin, CTGF expression, and facilitating crosstalk between macrophages and fibroblasts. Our findings suggest a promising strategy to prevent the progression of metabolic CKD by promoting the excretion of succinate in urine and/or using selective antagonists for SUCNR1.

**Supplementary Information:**

The online version contains supplementary material available at 10.1186/s12964-024-01481-5.

## Introduction

Chronic kidney disease (CKD) is characterized by a decline in kidney function, indicated by a glomerular filtration rate (GFR) of less than 60 mL/min per 1·73 m^2^, or the presence of kidney damage markers, or both, persisting for a minimum of 3 months, diminishing the patient’s quality of life and causing premature death [1]. CKD progresses to tubulointerstitial fibrosis eventually, regardless of various pathogenic factors [2]. The primary pathological process of renal interstitial fibrosis can be briefly and artificially divided into four overlapping phases: namely, priming (changes in the tissue microenvironment caused by kidney injury), activation (activation and proliferation of myofibroblasts), execution (massive extracellular matrix production and deposition), and progression (tubular atrophy and microvascular rarefaction), macrophages play an essential role in the priming phase [3].

Tubular epithelial cells are the most vulnerable intrinsic renal cells and can produce multiple chemokines, which promote blood monocyte recruitment into the injured kidney [4]. Monocytes can be differentiated into various macrophage phenotypes categorized as classical M1-type activation and alternative M2-type activation because of their heterogeneity and plasticity [4]. Numerous studies and clinical evidence suggested that activated M2 macrophages contributed to interstitial fibrosis [5–10]. Different groups have proved that IL-4, IL-13 [11], and TGFβ1 [12] were classical M2 polarization-inducing factors.

Besides, recent literature reported that succinate could promote macrophages alternatively activation via succinate receptor 1(SUCNR1), except for being a simple metabolic intermediate [13]. Succinate is an intermediate metabolite of the tricarboxylic acid cycle or a catabolic metabolite of microbial oligo−/polysaccharide fermentation [14]. Under normal physiological conditions, the plasma succinate might be in the range of 5–200 μM [15–17]. However, a growing body of literature reported persistent elevation of circulating succinate in chronic pathological conditions, including type 2 diabetes (T2D), obesity [15], and nonalcoholic fatty liver disease (NASH) [18]. Meanwhile, orphan G protein coupled-receptor 91(GPR91) was recently considered as SUCNR1 [19]. SUCNR1 was widely expressed throughout the body [20], including various immune cells, such as immature dendritic cells and macrophages [21]. Different teams have demonstrated that succinate-SUCNR1 regulated tumor-associated macrophages [22] and adipose-tissue-resident macrophages M2 type polarization [23].

The pathogenesis of renal interstitial fibrosis is highly complex, and current treatments remain insufficient. Therefore, the current study has examined the potential causal role of succinate in metabolic renal interstitial fibrosis and its ability to induce renal fibrosis by activating macrophages’ M2 phenotype.

## Methods

### Animal experiments

Male C57/BL6 mice (6-8w) were obtained from Sun Yat-Sen University and were kept in a temperature (25 °C) and humidity-controlled room with a 12:12-h light-dark cycle. All animal procedures were approved by the Animal Care and Use Committee of Sun Yat-Sen University. All animals were randomly assigned to the control group (*n* = 5) and the succinate group(n = 5). All mice were free to eat and drink during the experiment, and the succinate group was fed with special water (supplementary with 4% succinate, dissolved in distilled water) for 12w, the control group was fed with distilled water, the water was freshly prepared and replaced every two days [24].

For depletion of macrophages, the macrophage scavenger clodronate liposomes (CL, F70101C, FormuMax, USA) were adopted and administrated 150 μl per intraperitoneal injection twice a week. The succinate group was injected with control liposomes.

### Semiquantitative analyses of fibrotic area in the kidney tissue

After the experiment, mice were killed to collect the kidneys, embedded in paraffin, continuously cut 3-μm thickness, and stained with Masson Trichrome and Sirius red kit (Solarbio LIFE SCIENCE, Beijing, China). For semiquantitative analysis, ten randomly selected 400X fields of kidney cortex for each mouse were analyzed with Image Pro plus 6.0, and the calculating average percentage of kidney fibrotic area was used to further analyze.

### Immunoblotting analysis

Whole kidney tissue was homogenized with lysis buffer supplement with 1 mmol/l PMSF and 1% protease inhibitor mixture (RIPA, P0013, Beyotime, China) on ice, then centrifuged at 15,000 rpm for 20 minutes at 4 °C. The supernatants were collected after centrifugation. The protein concentration was measured and calibrated with the BCA protein assay kit (Thermo Scientific, Waltham, MA). For murine macrophage RAW 264.7 cells and rat renal fibroblast NRK-49F cells, the medium was discarded, and the cells were also lysed with the 1x SDS sample buffer after treatment. An equal amount of total protein was separated by SDS-PAGE (8,10,12%). All protein bands were quantified by Image ProPlus 6.0 software.

### Antibodies

Rabbit anti-Fibronectin (BA1772; BOSTER), Rabbit anti-α-SMA(BM3902; BOSTER), Rabbit anti-F4/80 (70,076 T;CST), Rabbit anti-CTGF(AF6582;Beyotime), Mouse anti-GAPDH(60004–1; Proteintech), Mouse anti-β-actin (sc1616; Santa Cruz), Mouse anti-β-tubulin(ab179513;Abcam), Rabbit anti-SUCNR1(NBP1-00861SS; Novus), Rabbit anti-β-catenin(51067–2-AP; Proteintech), Rabbit anti-non-p-β-catenin(8814S(Ser33/77/Thr41);CST), Rabbit anti-p-Akt(Ser473, 9271;CST) Rabbit anti-Akt(4691;CST), Rabbit anti-p-GSK3β(Ser9, 9323;CST), Rabbit anti-GSK3β(9832;CST), Rabbit anti-p-LRP6(2568;CST).

### Real-time PCR

Total RNAs from kidneys were extracted using Trizol reagent (Invitrogen), dissolved in RNAase-free water, measured by (Nanodrop 2000, Thermo Scientific), and a total amount of 1200 ng RNA was used to synthesize cDNA with Evo M-MLV RT Premix kit (AG11706, Accurate Biology, Changsha, China). The cDNAs were diluted for quantitative RT-PCR using (AG11704, Changsha, China) and (BIO-RAD, CFX96 touch). The cell samples were processed by application of EZ-press Cell to cDNA Kit (EZBioscience) following treatment. The primers used above were purchased from (TIANYIHUIYUAN, China). For more details, please see Supplementary Table 1.

### Immunohistochemistry staining

The kidneys were paraffin-embedded and cut into 3-μm sections. The sections were de-waxed, followed by antigen retrieval by boiling in 0.01 M citrate buffer (pH 6.0) for 2 min. After blocking with Goat serum for 1 h, the slides were incubated for 16 h at 4 °C with the primary antibody F4/80 (1:100; CST, 70076), followed by incubation with HRP-conjugated secondary antibody for 1 h at room temperature and then stained with the DAB kit. The sections were counterstained with hematoxylin and photographed through a digital pathology slide scanner (KFBIO).

### Detection of proliferation

Digested by trypsin, resuspension by DMEM/F12 medium, a density of 8 × 10^3^ NRK-49F cells /well were seeded into a 96-well plate and grown in a 37 °C cell culture incubator for 24 h. Following treatment, the supernatants were abandoned, washed with fresh DMEM/F12 twice, and replaced with 100 μL fresh DMEM/F12 with the addition of 10 μL/well CCK8 reagent (APExBIO, Houston, USA), gently mixed well, incubated in darkness and read the absorbance value at 450 nm. The relative survival rate was calculated by subtracting the background and normalizing the untreated group. For the EdU cell proliferation assay, a density of 2 × 10^4^ cells/well was seeded onto cell slides and placed into a 12-well plate. After administration, cell proliferation was detected by the EdU kit (Sangon Biotech, Shanghai, China) according to the manufacturer’s instructions (scanned with Olympus BX63, Japan).

### SUCNR1 siRNA transfection

RAW 264.7 were seeded into a 12-well plate and grew to 70%.

The cells were transfected with siRNA against mouse SUCNR1 or scramble siRNA (from Ruibo Biotech, Guangzhou) for 36 h.

### Cell culture and treatment

RAW 264.7 cells (ATCC) were cultured in DMEM containing 10% (vol/vol) FBS (Gibco, Thermo Fisher Scientific, American) and 1% (vol/vol) antibiotics (100 U/ml penicillin) at 37 °C in 5% CO2. For succinate treatment, 500 μM succinate (dissolved in distilled water) was added to the culture medium and incubated for 24 h or 48 h after starvation with DMEM FBS-free medium. To block β-catenin signaling, RAW 264.7 cells were pretreated with ICG-001(HY-14428, MCE, USA, 2 μM) for 1 h and then were treated with 500 μM succinate for various d. NRK-49F cells were cultured in DMEM /F12 containing 10% (vol/vol) FBS (Gibco, Thermo Fisher Scientific, American) and 1% (vol/vol) antibiotics (100 U/ml penicillin) at 37 °C in 5% CO2. For the conditioned medium administration, the prepared culture solution (CM: DMEM/F12 = 2:3) was mixed well and incubated for 48 h. NRK-49F cells (ATCC) were incubated for a conditioned medium following preincubation of anti-CTGF (A2042, Selleck, USA, 10 mg/ml) for 2 h.

### Statistical analyses

Values are presented as mean ± SEM. ANOVA and Bonferroni t-tests were used for statistical analysis by SPSS 26, and *P* < 0.05 was were considered significant.

## Results

### Succinate induces renal interstitial fibrosis in normal mice

**Fig. 1:**
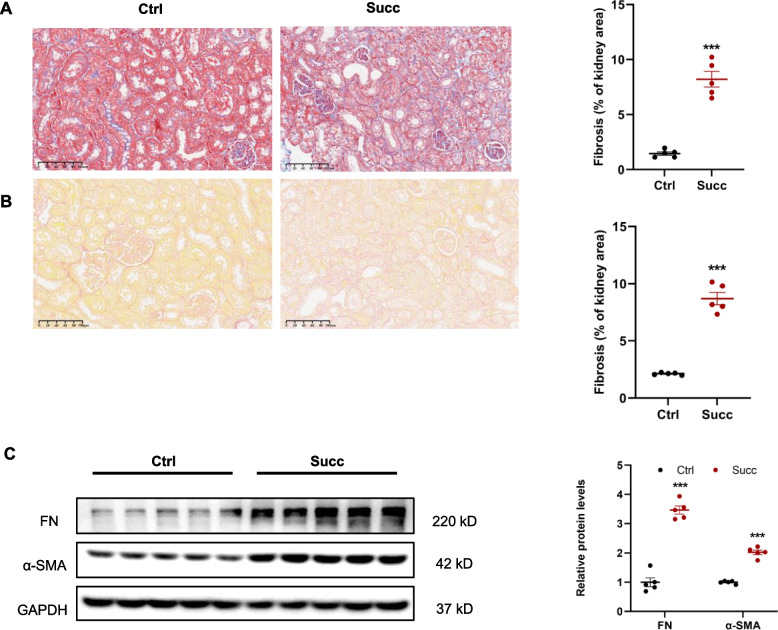
Succinate induced renal interstitial fibrosis in mice. Male C57 BL/6 mice were fed with special water (containing 4% succinate) for 12w. **A** Masson and Sirius red. **B** staining showed succinate induced renal interstitial fibrosis in mice.***P < 0.001 versus control group, *n* = 5. **C** Immunoblotting showed succinate increased renal protein levels of fibronectin and α-SMA. ***P < 0.001 versus control group, *n* = 5

We first identified whether succinate, as a causative factor, directly caused renal interstitial fibrosis. Quantitative Masson staining revealed succinate-induced interstitial fibrosis after 12 weeks of succinate treatment (Fig. 1A). The results of Sirius red staining consistently aligned with the Masson staining findings (Fig. 1B). Interestingly, there were no significant differences in glomerular mesangial expansion and glomerulosclerosis between the two groups. In addition, the protein expressions of fibrosis markers-fibronectin and α-SMA were analyzed by immunoblotting, and the results showed that succinate significantly increased renal fibronectin and α-SMA protein expressions (Fig. 1C). Collectively, quantitative staining and protein expression results demonstrated that succinate induced renal interstitial fibrosis.

### Succinate stimulates activation of profibrotic M2 phenotype and upregulation of profibrotic factors

**Fig. 2:**
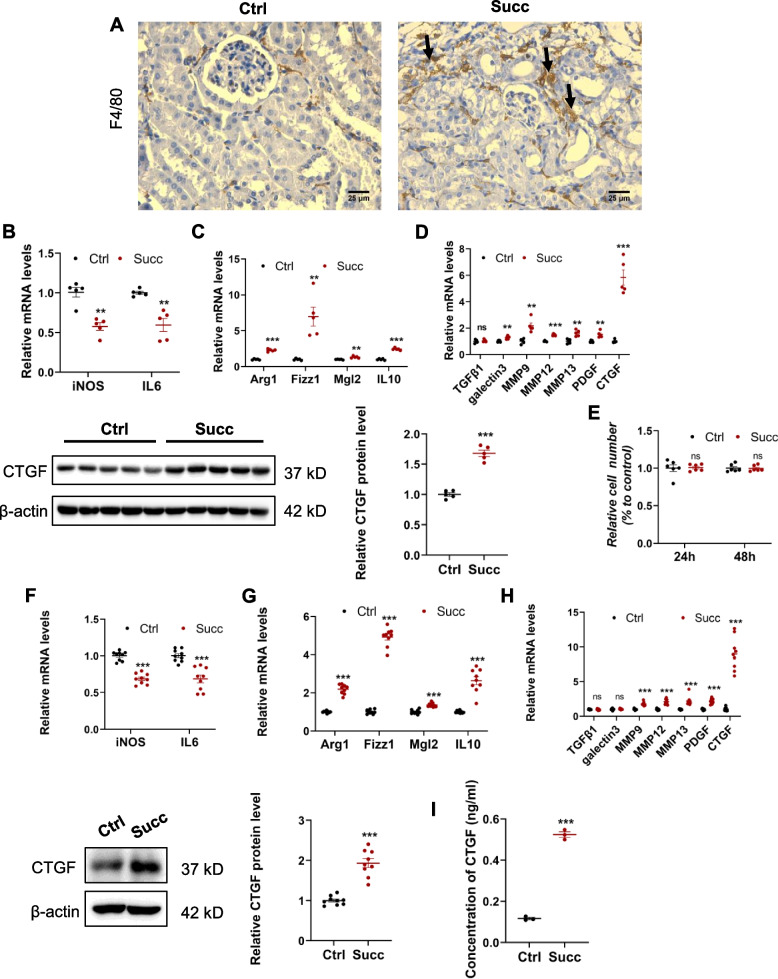
Succinate stimulated macrophage infiltration, activation of profibrotic M2 phenotype, upregulation of profibrotic factors in vivo and in vitro. **A** F4/80 immunohistochemistry staining indicated succinate markedly increased renal macrophage in the kidney interstitium rather than glomerulus. ***P < 0.001, versus control group, n = 5. **B** Renal proinflammatory M1 cytokines, including iNOS and IL6 mRNA levels, were reduced by succinate. **P < 0.01, versus the control group, n = 5. **C** The mRNA levels of anti-inflammatory M2 cytokines (Arg1, Fizz1, Mgl2, and IL-10) in the kidney were markedly increased. **P < 0.01;***P < 0.001, versus control group, n = 5. **D** Succinate upregulated renal M2 macrophages-related profibrotic factors expression (galectin3, MMP9, MMP12, MMP13, PDGF, and CTGF). ns, not significant; **P < 0.01;***P < 0.001, versus control group, n = 5. RAW 264.7 cells were treated at 500 μM succinate for 24 h, and quantitative PCR analysis and immunoblotting were adopted to detect the effects of succinate on M2 polarization and expression of profibrotic factors in vitro. **E** Succinate had no effects on the cell viability of RAW 264.7. Succinate downregulated M1 cytokines (iNOS and IL6) mRNA levels (**F**) while significantly upregulated M2 cytokines (Arg1, Fizz1, Mgl2, and IL-10) (**G**). ***P < 0.001, versus control group, *n* = 3, biologically repeated 3 times. Likewise, succinate remarkably increased M2 macrophage-related profibrotic factor expression (MMP9, MMP12, MMP13, PDGF, and CTGF) (**H**). ns, not significant; ***P < 0.001, versus control group, n = 3, biologically repeated 3 times. The cell supernatant was measured by mouse CTGF ELISA kit according to the manufacturer’s instructions (E-EL-M0340; Elabscience, Bethesda, MD). Similarly, succinate increased CTGF release of macrophages (**I**)

As reported, M2 macrophages played important profibrotic roles in renal fibrogenesis [5–10], we assumed whether succinate induced renal interstitial fibrosis by activating profibrotic M2 macrophages. Compared with the control group, the immunohistochemistry results of mice macrophage marker F4/80 revealed that succinate stimulated macrophage infiltration in the interstitial while having no obvious effects on the glomerulus (Fig. 2A). Besides, the renal mRNA levels of M1/Th1 markers (iNOS, IL6) in the succinate-treated group were lower than the control group (Fig. 2B). Meanwhile, the mRNA levels of M2/Th2 markers, including Arg1, Fizz1, Mgl2, and IL10, were remarkably higher than the control group (Fig. 2C). The mRNA and protein expressions of profibrotic factors including galectin3, MMP9, MMP12, MMP13, Platelet-derived growth factor (PDGF), Connective tissue growth factor (CTGF) was significantly enhanced in the kidney of the succinate group except for TGFβ1 (Fig. 2D).

We adopted RAW 264.6 cell line to validate further the activation of M2 polarization and upregulation of profibrotic factors in vitro.

The CCK8 results indicated that succinate did not affect the cell viability of RAW 264.7 cells (Fig. 2E). Next, the mRNA levels of M1/Th1 markers (iNOS, IL6) were decreased (Fig. 2F), and M2/Th2 markers (Arg1, Fizz1, Mgl2, IL10) in RAW 264.7 were increased (Fig. 2G) by succinate treatment. A series of profibrotic factors (MMP9, MMP12, MMP13, PDGF, and CTGF) in RAW 264.7 were also apparently upregulated by succinate (Fig. 2H). The release of CTGF in macrophages was also enhanced by succinate (Fig. 2I). Additionally, the results from succinate-treated bone marrow-derived macrophages further validated the aforementioned findings (Supplementary Fig. 1). These in vitro and in vivo results above collaboratively demonstrated that succinate could stimulate macrophage M2 polarization and the expression of profibrotic factors.

### Depletion of macrophage ameliorates succinate-induced interstitial fibrosis

**Fig. 3:**
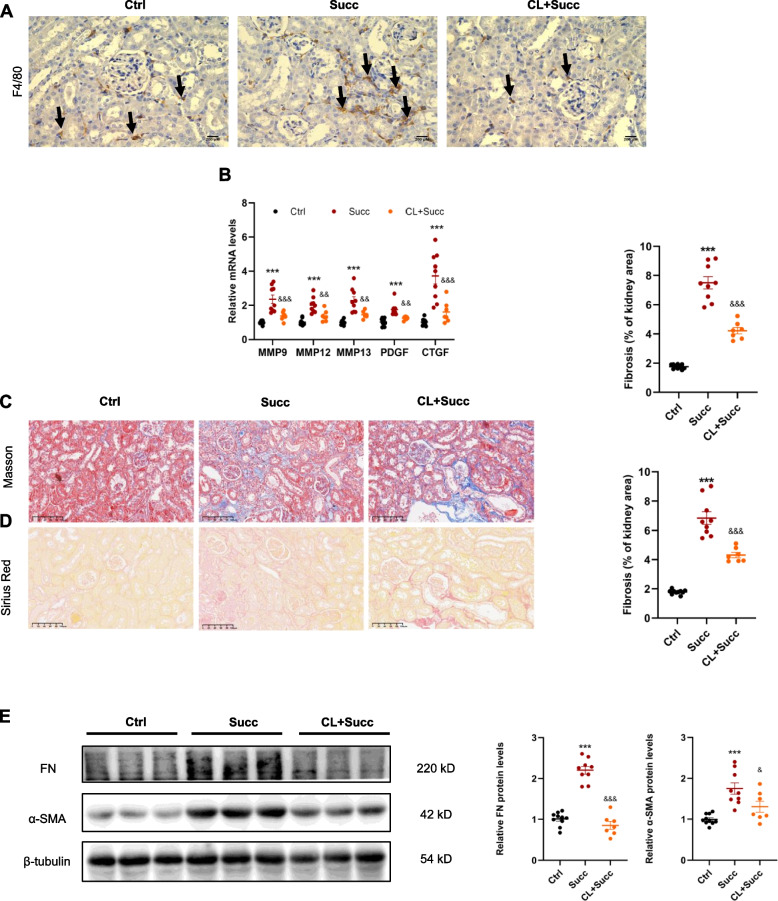
Macrophage removal alleviated succinate-induced kidney interstitial fibrosis. **A** F4/80 immunohistochemistry staining showed clodronate liposomes effectively removed kidney interstitial macrophages of mice. **B** Clodronate liposomes markedly prevented succinate-mediated upregulation of M2-related profibrotic factors in the kidney. ***P < 0.001, versus control group; &&&*P* < 0.001, versus succinate group, *n* = 10 in the control group, *n* = 9 in succinate group and *n* = 7 in clodronate liposomes group. **C** Masson and Sirius Red staining (**D**) revealed succinate-induced interstitial fibrosis was alleviated by clodronate liposomes. ***P < 0.001, versus control group; &&&*P* < 0.001, versus succinate group, n = 10 in control group, n = 9 in succinate group and *n* = 7 in clodronate liposomes group. **E** The elevation of protein levels of renal fibronectin and α-SMA was revised by clodronate liposomes. ***P < 0.001, versus control group; &&&*P* < 0.001, &*P* < 0.05, versus succinate group, *n* = 10 in control group, *n* = 9 in succinate group and *n* = 7 in clodronate liposomes group

To classify the critical role of macrophages in succinate-induced renal fibrosis, macrophage scavenger clodronate liposomes were administrated intraperitoneally for mice [25, 26]. As expected, clodronate liposomes effectively depleted renal macrophages (Fig. 3A). The mRNA levels of M2 macrophage-produced profibrotic factors were increased by succinate while significantly inhibited by clodronate liposomes treatment (Fig. 3B). Accordingly, the Masson (Fig. 3C) and Sirius red staining (Fig. 3D) indicated that clodronate liposomes effectively ameliorated succinate-induced interstitial fibrosis. The succinate-increased protein levels of fibronectin and α-SMA also decreased (Fig. 3E). The above in vivo results demonstrated that M2 macrophages were involved in succinate-induced fibrogenesis.

### Activated macrophages promote the proliferation and activation of renal fibroblast via paracrine action

Myofibroblasts are usually considered to be the predominant effector cells in renal fibrosis, and renal fibroblasts are the classical cell source of myofibroblasts [27]. Firstly, we used quantitative RT-PCR and immunoblotting to validate the SUCNR1 mRNA and protein expression of cultured renal interstitial fibroblasts (NRK-49F), and SD rat kidney with abundant SUCNR1 was used as a positive control. Compared with the rat kidney, the SUCNR1 Ct value of NRK-49F was undetectable, and there was no visible protein band at the SUCNR1 position (Supplemental Fig. 2A). Subsequently, the direct effects of succinate on proliferation and activation in the NRK-49F were detected by CCK8, Edu, and western blot. Cell viability and proliferation were unchanged between the control and succinate groups (Supplemental Fig. 2B). In addition, the protein levels of two hallmarks of fibroblast activation, fibronectin, and α-SMA, were not increased by succinate (Supplemental Fig. 2C). These results showed that succinate had no direct effects on the proliferation and activation of NRK-49F.

**Fig. 4:**
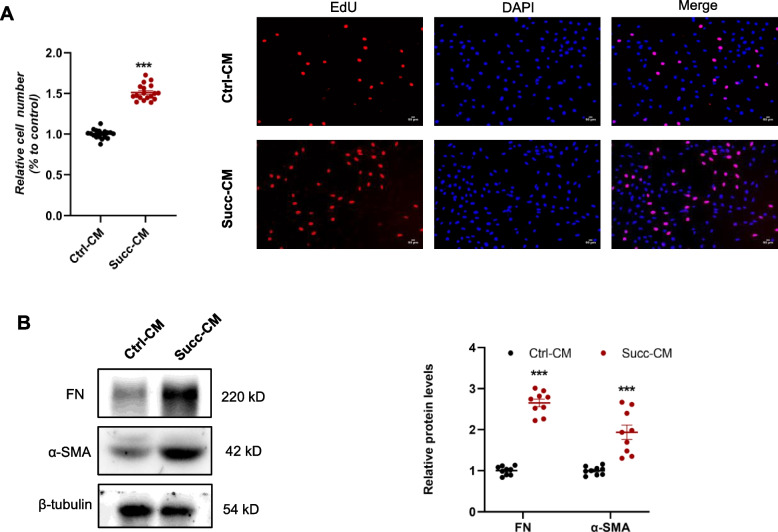
Conditioned medium of macrophages following succinate treatment triggered renal fibroblast proliferation and activation. 500 μM succinate was used to stimulate RAW 264.7 cells for 48 h, and the conditioned medium was collected, centrifuged, and incubated with NRK-49F. **A** The results of the CCK8 assay and EdU staining displayed that the conditioned medium of the succinate group enhanced NRK-49F proliferation. ***P < 0.001, versus control group, *n* = 6 in CCK8 and *n* = 3 in EdU staining, biologically repeated 3 times. **B** Protein levels of fibronectin and α-SMA were increased by the conditioned medium of the succinate group. ***P < 0.001, versus control group, *n* = 3, biologically repeated 3 times

As shown above, succinate promoted a series of profibrotic factors mRNA and protein expression, especially growth factors (PDGF [28] and CTGF [29]) which robustly stimulated renal fibroblast proliferation and activation. We collected the conditioned medium of RAW 264.7 after succinate administration, centrifuged, and stimulated NRK-49F. The results showed that the succinate-CM enhanced NRK-49F viability and proliferation compared with the ctrl-CM group (Fig. 4A). The protein levels of fibronectin and α-SMA were dramatically upregulated by the succinate-CM as well, indicating the activation of fibroblasts (Fig. 4B). The succinate-CM that derived from BMDMs exhibited comparable stimulatory effects on NRK-49F (Supplementary Fig. 3). These results remained that succinate mediated the proliferation and activation of renal fibroblasts through indirect paracrine action rather than direct effects.

### Succinate-SUCNR1 is involved in macrophage M2 polarization, upregulation of profibrotic factors, and paracrine effects on fibroblasts

**Fig. 5:**
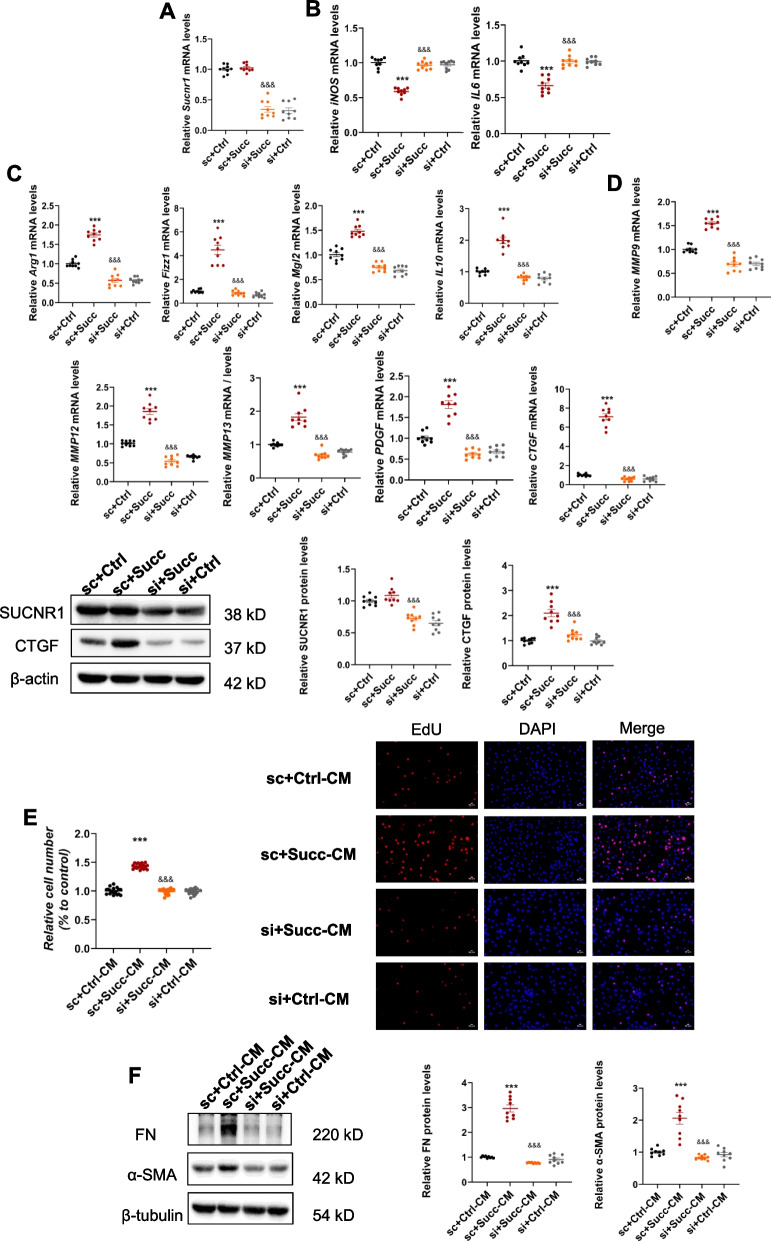
SUCNR1 was required for the effects of succinate on M2 polarization, upregulation of profibrotic factors, and paracrine actions on fibroblasts. RAW 264.7 was transfected with SUCNR1 siRNA for 36 h, and 500 μM succinate was stimulated for 24 h. **A** SUCNR1 mRNA levels were significantly reduced by SUCNR1 siRNA. &&&*P* < 0.001, versus succinate group, *n* = 3, biologically repeated 3 times. **B** Knockdown of SUCNR1 inhibited the downregulation of proinflammatory M1 cytokines (iNOS and IL6) and upregulation of anti-inflammatory M2 cytokines (Arg1, Fizz1, Mgl2, IL10) (**C**) induced by succinate. ***P < 0.001, versus the control group, &&&*P* < 0.001, versus the succinate group, *n* = 3, biologically repeated 3 times. **D** The elevated profibrotic factors were also restored by SUCNR1 siRNA. ***P < 0.001, versus the control group, &&&*P* < 0.001, versus the succinate group, *n* = 3, biologically repeated 3 times. **E** The proliferative effects of the conditioned medium in the succinate group were abolished by SUCNR1 siRNA. ***P < 0.001, versus the control group, &&&*P* < 0.001, versus the succinate group, *n* = 6, biologically repeated 3 times. **F** The enhanced protein expressions of fibronectin and α-SMA were reduced by SUCNR1 siRNA. ***P < 0.001, versus the control group, &&&*P* < 0.001, versus the succinate group, *n* = 3, biologically repeated 3 times

SUCNR1 was a specialized receptor for succinate [19] and also expressed on macrophages [21]. siRNA transfection against mouse SUCNR1 for RAW 264.7 was applied to verify the necessity for SUCNR1 on these effects of succinate. Figure 5A and D showed that the SUCNR1 siRNA markedly lowered the mRNA and protein levels of SUCNR1 in RAW 264.7 (Fig. 5A, D). Consistent with previous reports [22], succinate-mediated macrophage M2 polarization was abrogated by SUCNR1 siRNA, as indicated by Fig. 5B and C. In addition, the succinate-increased profibrotic factors were also reduced by SUCNR1 siRNA (Fig. 5D). Knockdown SUCNR1 inhibited the paracrine effects (proliferation and activation) of RAW 264.7 on NRK-49F (Fig. 5E, F). These results suggested that succinate stimulated M2 polarization, upregulation of profibrotic factors of macrophage, proliferation, and activation of fibroblasts through the SUCNR1.

### CTGF played a significant role in the stimulation effects on fibroblasts

**Fig. 6:**
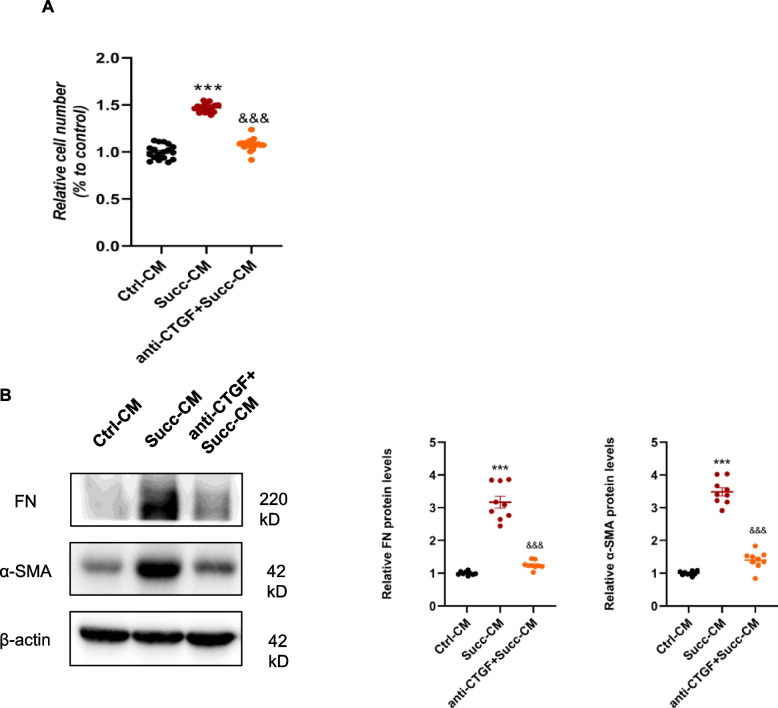
CTGF neutralizing antibody inhibited the stimulation of fibroblasts by macrophage-conditioned medium. **A** CTGF antibody prevented the proliferative effects of CM on NRK-49F, indicated by the results of the CCK8 assay. ***P < 0.001, versus control group, n = 6 in CCK8, biologically repeated 3 times. **B** Also, the CTGF antibody suppressed the activation effects of CM on NRK-49F, as indicated by the results of the protein quantitative analysis of fibronectin and α-SMA. ***P < 0.001, versus control group, &&&*P* < 0.001, versus the succinate group, n = 3, biologically repeated 3 times

Numerous scientific studies and clinical data have demonstrated that CTGF is involved in the pathogenesis of kidney fibrosis [30–33], and among the various profibrotic factors, CTGF was the most significantly upregulated by succinate. To investigate the role of CTGF on fibroblast stimulation, the anti-CTGF antibody was pretreated for NRK-49F for 2 h before incubation of the conditioned medium. Anti-CTGF antibody restrained cell proliferation stimulated by the conditioned medium (Fig. 6A). The conditioned medium-mediated activation of NRK-49F was substantially suppressed by an anti-CTGF antibody (Fig. 6B). These results demonstrated that among the various profibrotic factors increased by succinate in RAW 264.7, CTGF was the most important and significantly affected NRK-49F.

### Succinate regulates CTGF expression through the activation of β-catenin

**Fig. 7:**
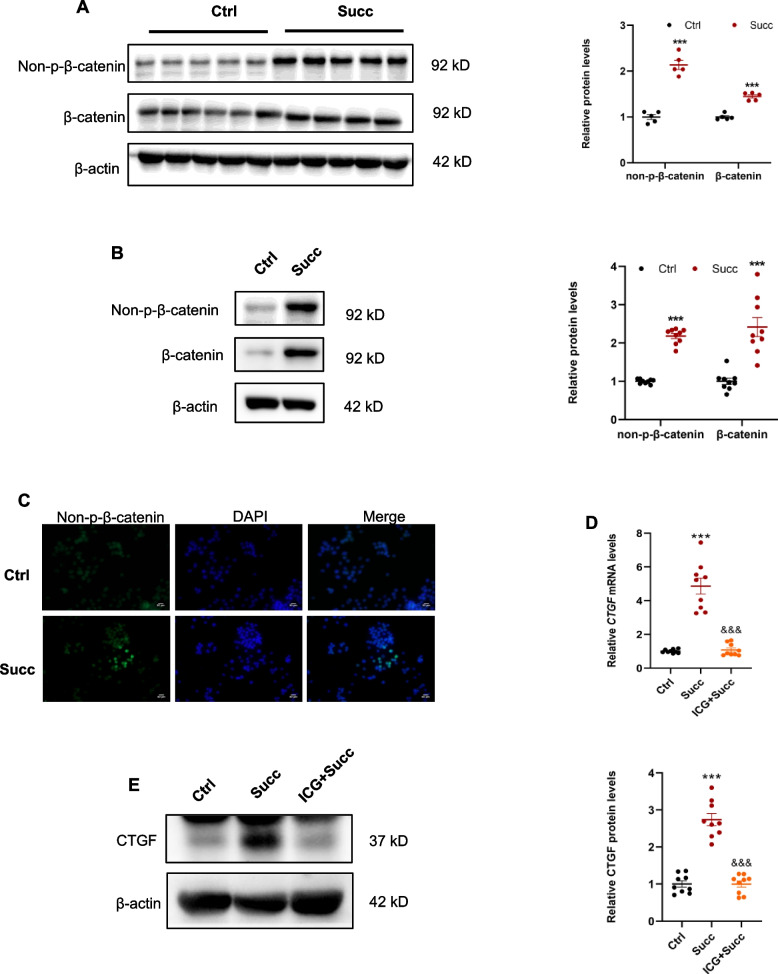
Succinate promoted CTGF expression through activation of β-catenin. **A** Succinate increased protein levels of non-p-β-catenin and β-catenin in the mice kidney. ***P < 0.001, versus control group, *n* = 5. RAW 264.7 was treated with 500 μM succinate for 12 h. **B** Succinate enhanced protein levels of non-p-β-catenin and β-catenin in the RAW 264.7. ***P < 0.001, versus control group, n = 3, biologically repeated 3 times. **C** Succinate promoted translocation of non-p-β-catenin into the nucleus. ICG-001 (2 μM) pretreatment RAW 264.7 for 1 h, 500 μM succinate stimulation for 24 h and 48 h. **D** ICG-001 prevented the increase of CTGF mRNA induced by succinate. ***P < 0.001, versus control group, &&&*P* < 0.001, versus the succinate group, n = 3, biologically repeated 3 times. **E** The elevation of CTGF protein level was also lowered by ICG-001. ***P < 0.001, versus control group, &&&*P* < 0.001, versus the succinate group, n = 3, biologically repeated 3 times

As results displayed above, CTGF was upregulated by succinate-SUCNR1 at the transcriptional levels in the RAW 264.7 cells. β-catenin of the macrophages promoted alternative macrophage activation and contributed to kidney fibrosis [9]. Besides, β-catenin was a critical transcription factor for CTGF [34, 35]. So, we proposed the hypothesis that succinate increased CTGF expression via activating the β-catenin. The immunoblotting results of mice kidneys revealed that the protein levels of non-p-β-catenin and β-catenin were remarkably increased (Fig. 7A). These results were further validated in vitro, indicated by the elevation of protein levels of non-p-β-catenin, β-catenin, and nuclear translocation of non-p-β-catenin in the RAW 264.7 (Fig. 7B, C). Transcriptional inhibitor of β-catenin signaling ICG-001 significantly abolished the boosted-mRNA and protein of CTGF (Fig. 7D, E). These outcomes suggested that succinate upregulated CTGF expression via the β-catenin.

### Succinate-SUCNR1 activated β-catenin signaling through p-Akt/p-GSK3β

As known, β-catenin is a classic participator in the canonical Wnt pathway. WNT ligands and p-LRP6 (a component of receptor complex) were required for canonical Wnt signaling activation [36]. We next explored whether succinate-SUCNR1 stimulated β-catenin signaling depending on the canonical Wnt way. The results of renal quantitative RT-PCR revealed that succinate had no stimulating effects on the mRNA levels of Wnt3a and Wnt5a (Supplementary Fig. 4) in vivo. Surprisingly succinate noticeably reduced the mRNA levels of Wnt3a and Wnt5a in the RAW 264.7 in vitro (Supplementary Fig. 5). Furthermore, it showed that succinate did not alter the protein levels of p-LRP6 in the kidney (Supplementary Fig. 6) and RAW 264.7 (Supplementary Fig. 7). These results implied that succinate activated β-catenin in a canonical Wnt pathway-independent manner.

**Fig. 8:**
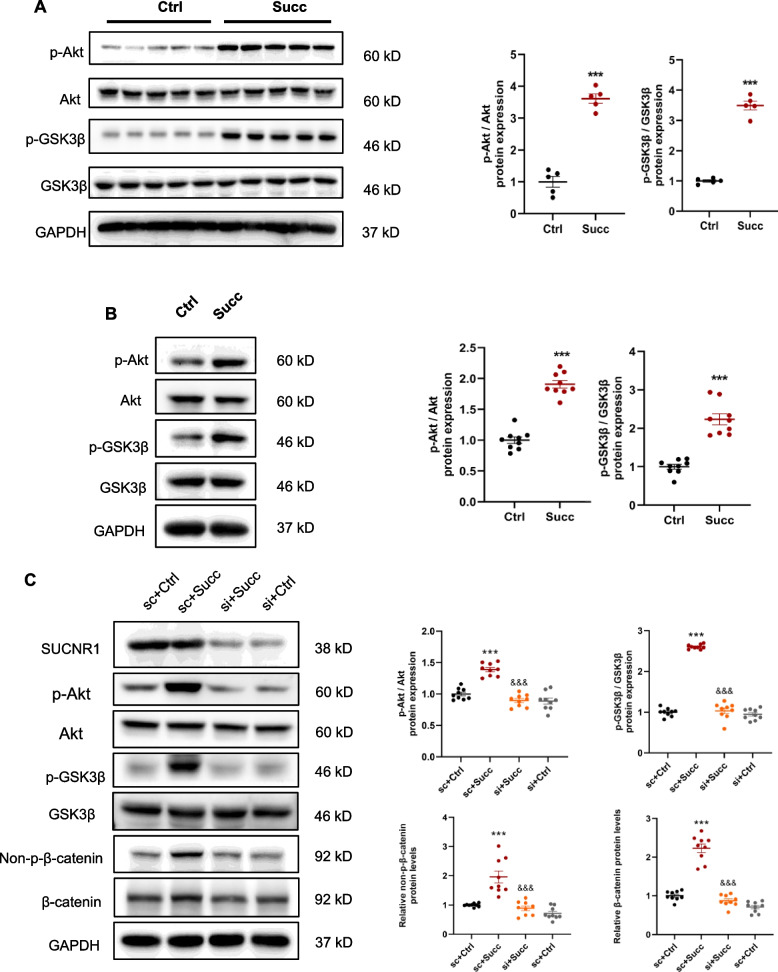
Succinate-SUCNR1 activated p-Akt/p-GSK3β, and β-catenin pathways. **A** Succinate increased p-Akt,p-GSK3β protein levels in the mice kidney. ***P < 0.001, versus control group, n = 5. RAW 264.7 was treated with 500 μM succinate for 12 h. **B** Succinate also increased p-Akt and p-GSK3β protein levels in the RAW 264.7. ***P < 0.001, versus control group, n = 3, biologically repeated 3 times. RAW 264.7 was transfected with SUCNR1 siRNA for 36 h, and 500 μM succinate was stimulated for 12 h. **C** SUCNR1 siRNA abrogated the activation of p-Akt, p-GSK3β, non-p-β-catenin, and β-catenin in the RAW 264.7. ***P < 0.001, versus scramble siRNA+control group, &&&*P* < 0.001, versus the scramble siRNA+succinate group, n = 3, biologically repeated 3 times

One component of the “destruction complex” was glycogen synthase kinase 3β (GSK3β), which was responsible for the phosphorylation of β-catenin, leading to ubiquitination and subsequently degrading in proteasomes [37]. When GSK3β was phosphorylated at Ser9 by p-Akt (Ser473), the ability of phosphorylation of β-catenin was lost, resulting in the accumulation of non-p-β-catenin in cytoplasm and translocation into the nucleus [38]. As expected, the protein ratios of p-Akt/Akt and p-GSK3β/ GSK3β increased in the kidney (Fig. 8A) and the RAW 264.7 cells (Fig. 8B). In addition, succinate triggered p-Akt/p-GSK3β and β-catenin signaling via activation of SUCNR1 as the knockdown of SUCNR1 could prevent the stimulating effects of succinate (Fig. 8C).

## Discussion

Serum succinate was elevated in patients with T2D, obesity [15], and NASH [18], it has been reported that succinate of the liver tissues was significantly increased in NASH mice. Excessive succinate derived from impaired succinate dehydrogenase (SDH) in the hepatocytes promoted hepatic stellate cells (HSCs) activation and extracellular matrix production in a SUCNR1-dependent way [39]. Simultaneously, another group proved that uncoupling protein 1 (UCP1) KO mice exhibited higher succinate levels of liver tissues than the wild-type mice as a result of diminished capacity to clear succinate from the circulation and succinate-SUCNR1 regulated activation in liver HSC populations, thus exacerbating the fibrosis progress of the non-alcoholic fatty liver disease [40].

However, there was no available research about the causative effects of succinate on the kidney. Our recently published study found that the serum succinate of mice was doubled following succinate administration compared with the control group [41], and succinate caused proximal tubular cell apoptosis. Notably, the present study was the first to reveal that succinate induces renal interstitial fibrosis through macrophage M2 polarization. Renal fibrosis is characterized by glomerulosclerosis and interstitial fibrosis. However, our study showed no evidence of glomerulosclerosis in the succinate-treated mice. This result suggests that succinate treatment causes specific interstitial fibrosis in the kidney. Furthermore, except for renal fibrosis, proteinuria is also a pathological feature of CKD, and our results showed that succinate promoted urinary albumin excretion (Supplementary Fig. 8). Taken together, our two studies revealed the damaging effect of succinate on the kidney, suggesting that succinate might be a causative factor of CKD.

How does the high level of succinate play a role in kidney damage? It is mainly due to the high expression of the succinate receptor in the kidney. Its receptor SUCNR1 was abundantly expressed on tubular epithelial cells of multiple segments, including proximal tubules, distal tubules [19], the cortical thick ascending limb (cTAL) of Henle’s loop, the macula densa (MD), and the cortical and medullary collecting duct (CD) [42]. Our recently published study showed that succinate caused proximal tubular cell apoptosis by SUCNR1 [41]. As it is known, tubular cells are the primary component of the kidney, thus we investigated their response. Firstly, succinate did not alter the mRNA expression of M1 and M2 markers related to macrophages, as well as profibrotic factors in HK2 cells (Supplementary Fig. 9). Secondly, injured tubule cells have the ability to secrete cytokines that promote fibroblast proliferation and activation. The conditioned medium from succinate-treated HK2 cells did not induce proliferation and activation in NRK-49F cells (Supplementary Fig. 10). Finally, we observed that succinate upregulated the mRNA expression of chemokines (MCP-1 and CX3CL1), and the conditioned medium from succinate-treated HK2 cells upregulated M1 markers (Supplementary Fig. 11). These results implied that injured tubule cells promote monocyte infiltration through chemokines and differentiate into M1 macrophages to clear cellular debris and maintain kidney homeostasis during the early phase of injury. As the injury progresses, succinate may polarize M1 macrophages into M2 macrophages, contributing to renal fibrosis. These results suggest that damaged tubular cells participate in the fibrosis process by mainly affecting macrophages in the succinate-induced renal fibrosis model. To validate the effects of succinate on macrophages, future experiments using macrophage-specific SUCNR1 knockout mice are necessary to address this critical limitation of the study. It is well known that tubular cells are responsible for reabsorbing proteins from the glomerular filtrate, the injured tubular cells caused the distribution of protein reabsorption, which may contribute to proteinuria [1]..Besides, we speculated podocytes were very likely to express SUCNR1, and succinate might cause damage to podocytes via SUCNR1 leading to proteinuria. These assumptions need more effort to testify. In our study, succinate treatment simultaneously caused tubular cell injury, proteinuria, and renal intestinal fibrosis, leading to CKD.

The accumulation of M2 macrophages closely correlates with renal fibrosis in human kidney diseases and animal models [5–10]. Over the years, evidence has accumulated that M2 macrophages promoted fibroblast proliferation and activation in renal fibrosis. Partially by secreting a number of molecules, including MMP2 [43], MMP9 [44], MMP12 [45], galectin 3 [46], PDGF, and CTGF [29]. In the present study, we have demonstrated that succinate-SUCNR1 induced renal macrophages M2 polarization and release of M2-related profibrotic factors, especially CTGF, stimulate fibroblast proliferation and activation, eventually leading to renal interstitial fibrosis. Our results further complement the molecular mechanism of macrophages in kidney fibrosis. It is also reported that tumor-derived succinate promoted tumor-associated macrophage (TAM) polarization and IL-6 release via SUCNR1, resulting in cancer metastasis [22]. Besides, succinate-SUCNR1 drove inflammation in the liver and promoted inflammatory pathogenesis [40]. Abundant accumulating succinate from macrophages activated by inflammatory signals in the synovial fluids from rheumatoid arthritis patients enhanced IL-1β production and release, perpetuating inflammation [47]. These findings from different diseases implied that the functions of succinate-SUCNR1 in the macrophages play a critical pathological role in inflammatory-related diseases.

Succinate had no directly stimulating effects on renal fibroblasts that did not express SUCNR1 regardless of proliferation or activation, demonstrating the necessity of SUCNR1 for succinate function. However, we uncovered a cell crosstalk between renal intestinal macrophages and fibroblasts. The results of the molecular mechanism studies for the first time showed that succinate upregulated macrophages CTGF transcription by activating β-catenin in a Wnt and p-LRP6-independent manner. Furthermore, succinate reduced the mRNA levels of Wnt3a and Wnt5a, which might serve as negative feedback for the WNT/β-catenin pathway. These findings greatly enriched downstream signaling pathways of succinate-SUCNR1.

The morbidity and mortality of chronic kidney disease are growing yearly due to the increasing prevalence of chronic metabolic diseases like diabetes mellitus, hypertension, and obesity [48]. Current therapies for CKD consist of renin-angiotensin system (RAS) blockade, mineralocorticoid receptor blockers, the endothelin 1 receptor antagonist, and the sodium-glucose transporter 2 (SGLT2) inhibitor. However, these treatment effects are limited as the onset of CKD is very elusive [2]. There is a great need to develop novel therapeutic approaches to stop or reverse progression at the early stages of CKD onset.

Succinate is elevated in peripheral circulation when alteration of cellular metabolism occurs with an insult or injury. Measuring the circulating succinate with a small volume of serum samples is easy and safe. Based on our study, the level of circulating succinate might be used as an early predictor of kidney injury. For patients with elevated succinate, reducing the production or promoting the excretion of succinate would effectively delay the progression of renal fibrosis. In addition, a high-affinity, human-selective antagonist for SUCNR1 denoted NF-56-EJ40 has been developed in 2019 [49], also providing a potential therapeutic target.

However, the current study also has several limitations. Firstly, the depletion of macrophages by clodronate liposomes is a well-recognized method to validate the role of macrophages in animal models [50]. While adopting bone marrow-specific SUCNR1 knockout mice would better confirm our findings. Secondly, samples of clinical CKD patients should be included and analyzed. As demonstrated, diabetes mellitus and obesity are important risk factors of CKD [2], At the same time, the serum succinate levels of diabetes and obesity patients are elevated^15^which is also observed in our model. These reports and results could reinforce the pathogenic capacity of succinate.

In summary, our study revealed that succinate functioned as a risk factor rather than a metabolic intermediate and induced renal interstitial fibrosis through activating profibrotic M2 macrophages.

CTGF played a significant role in the crosstalk between the macrophages and fibroblasts. Mechanically, succinate-SUCNR1 mediated CTGF transcription by a p-Akt/p-GSK3β/β-catenin pathway which was Wnt and p-LRP6 independent. Our findings provide a foundation for future prevention and treatment of metabolic CKD (Supplementary Fig. 12).

### Supplementary Information

**Additional file 1: Supplementary Fig. 1.** Succinate stimulated activation of profibrotic M2 phenotype, upregulation of profibrotic factors in Bone marrow-derived macrophages. **Supplementary Fig. 2.** Succinate had no directive effects on NRK-49F. **Supplementary Fig. 3.** Conditioned medium of BMDMs following succinate treatment triggered renal fibroblast proliferation and activation. **Supplementary Fig. 4.** Succinate had no significant stimulatory effects on renal Wnt3a and Wnt5a. **Supplementary Fig. 5.** Succinate reduced mRNA levels of Wnt3a and Wnt5a in the macrophage. **Supplementary Fig. 6.** Succinate had no significant stimulatory effect on renal tissue p-LRP6. **Supplementary Fig. 7.** Succinate had no significant stimulatory effect on p-LRP6 of macrophage. **Supplementary Fig. 8.** Succinate caused mice proteinuria. **Supplementary Fig. 9.** Succinate did not change the mRNA expressions of macrophages-related M1, M2 markers, and profibrotic factors in HK2 cells. **Supplementary Fig. 10.** Succinate treated-HK2 cells failed to enhance the proliferation and activation of NRK-49F fibroblast. **Supplementary Fig. 11.** Conditioned medium of HK2 cells following succinate treatment induced macrophages adopting pro-inflammatory M1 polarization. **Supplementary Fig. 12.** The overview of succinate-SUCNR1 in renal fibrosis.

**Additional file 2.**
