# Supplementary material for: The pathogenic role of succinate-SUCNR1: a critical function that induces renal fibrosis via M2 macrophage
Source: Cell Commun Signal. 2024 Jan 30;22:78. doi: 10.1186/s12964-024-01481-5 (PMC10826041; doi:10.1186/s12964-024-01481-5)
Supplement: Supplementary file 1 — Additional file 1: Supplementary Fig. 1. Succinate stimulated activation of profibrotic M2 phenotype, upregulation of profibrotic factors in Bone marrow-derived macrophages. Supplementary Fig. 2. Succinate had no directive effects on NRK-49F. Supplementary Fig. 3. Conditioned medium of BMDMs following succinate treatment triggered renal fibroblast proliferation and activation. Supplementary Fig. 4. Succinate had no significant stimulatory effects on renal Wnt3a and Wnt5a. Supplementary Fig. 5. Succinate reduced mRNA levels of Wnt3a and Wnt5a in the macrophage. Supplementary Fig. 6. Succinate had no significant stimulatory effect on renal tissue p-LRP6. Supplementary Fig. 7. Succinate had no significant stimulatory effect on p-LRP6 of macrophage. Supplementary Fig. 8. Succinate caused mice proteinuria. Supplementary Fig. 9. Succinate did not change the mRNA expressions of macrophages-related M1, M2 markers, and profibrotic factors in HK2 cells. Supplementary Fig. 10. Succinate treated-HK2 cells failed to enhance the proliferation and activation of NRK-49F fibroblast. Supplementary Fig. 11. Conditioned medium of HK2 cells following succinate treatment induced macrophages adopting pro-inflammatory M1 polarization. Supplementary Fig. 12. The overview of succinate-SUCNR1 in renal fibrosis. [file 12964_2024_1481_MOESM1_ESM.pdf]

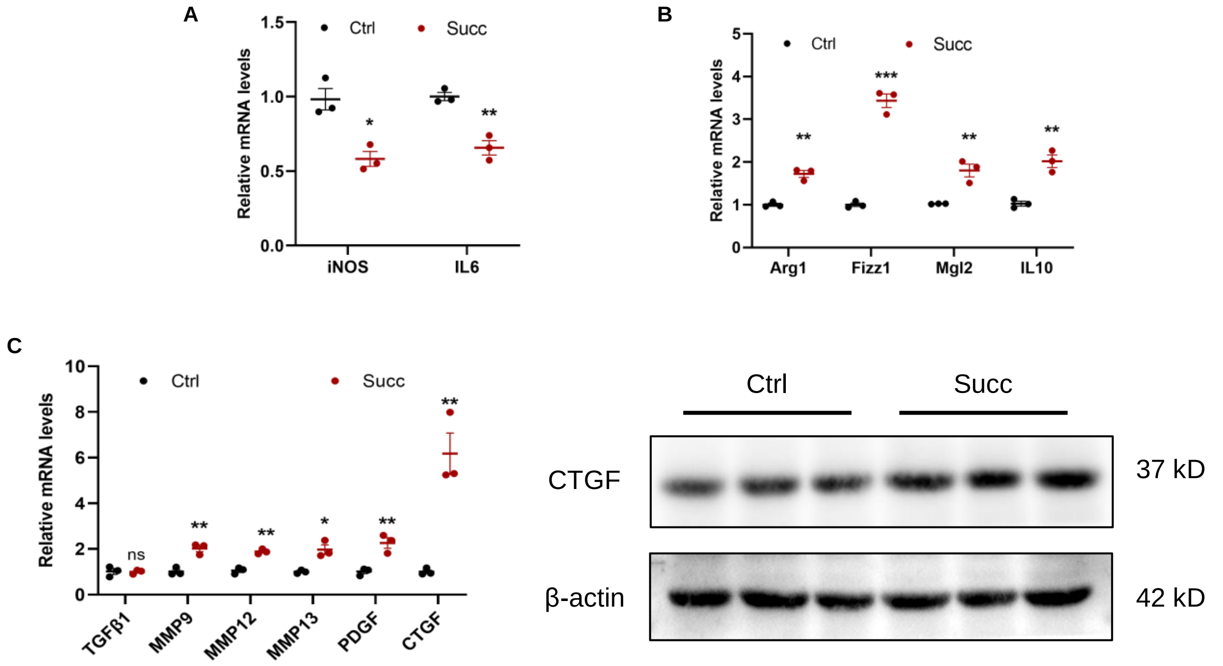

Supplementary Figure 1 Succinate stimulated activation of profibrotic M2 phenotype, upregulation of profibrotic factors in Bone marrow-derived macrophages

(A-C) BMDMs were also treated at 500μM succinate for 24h, and quantitative PCR analysis and immunoblotting were adopted to detect the effects of succinate on M2 polarization and expression of profibrotic factors. ns>0.05, *P<0.05, **P<0.01, ***P<0.001, versus the control group, n=3.

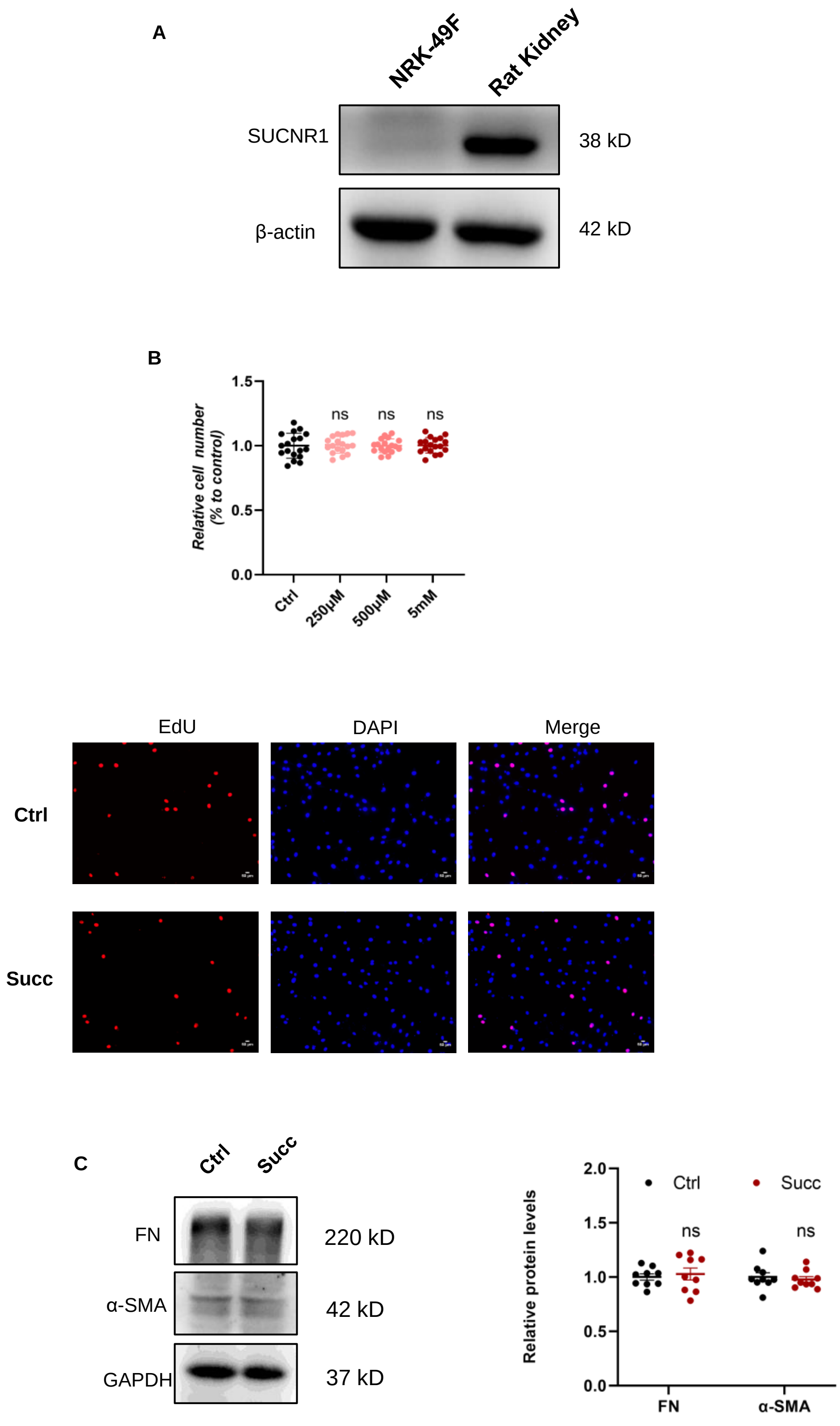

Supplementary Figure 2 Succinate had no directive effects on NRK-49F

(A) Validation of SUCNR1 protein expression in NRK-49F by immunoblotting. SD rat kidney was used for positive control. A range of 250μM and 5mM succinate was treated for NRK-49F for 48h.

(B) The results of the CCK8 assay and EdU staining demonstrated that succinate had no effects on proliferation. ns>0.05, versus the control group, n=6 in CCK8 and n=3 in EdU staining, biologically repeated 3 times.

(C) Succinate could not trigger the elevation of fibronectin and α-SMA. ns>0.05, versus the control group, n=3, biologically repeated 3 times.

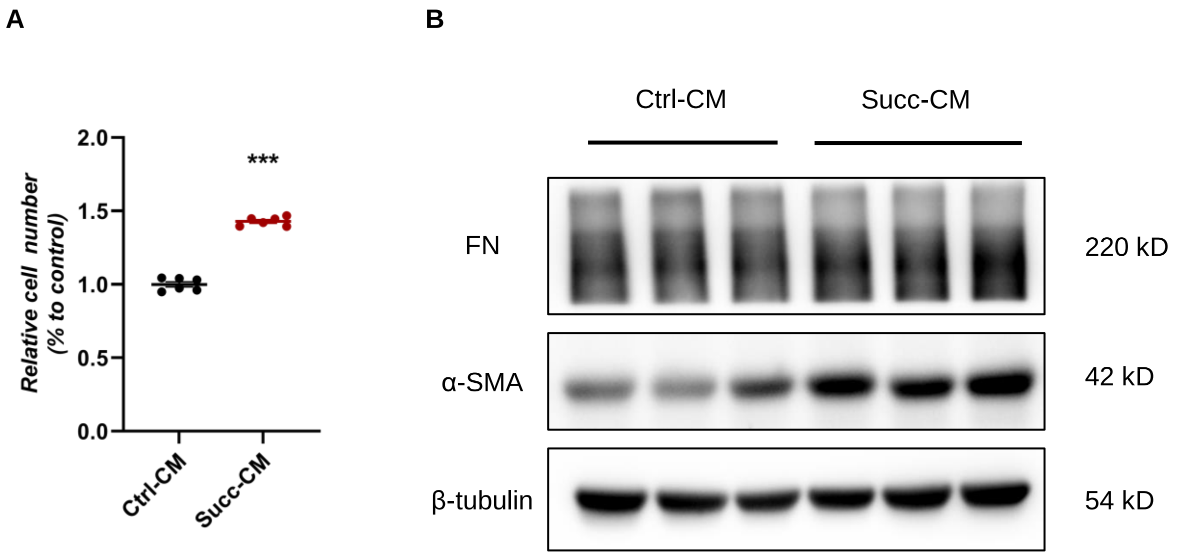

Supplementary Figure 3 Conditioned medium of BMDMs following succinate treatment triggered renal fibroblasts proliferation and activation

500μM succinate was used to stimulate BMDMs cells for 48h, and the conditioned medium was collected, centrifuged, and incubated with NRK-49F cells. The proliferation and activation of fibroblasts were detected by CCK8 assay and WB individually. ****P*<0.001, versus the control group, n=3 or 6.

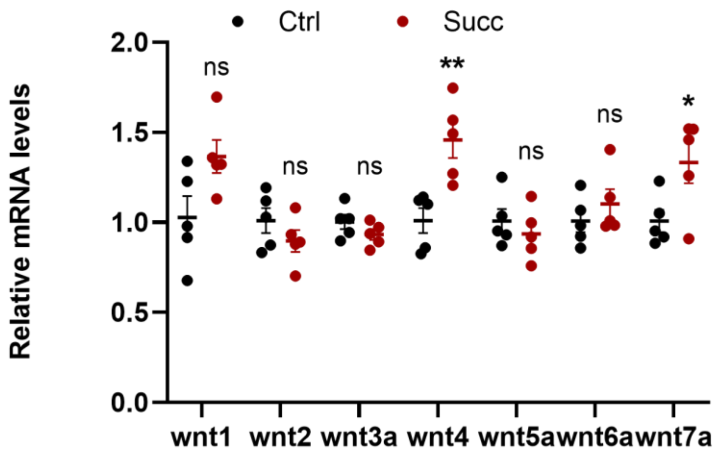

Supplementary Figure 4 Succinate had no significant stimulatory effects on renal Wnt3a and Wnt5a

Succinate had no significant stimulatory effects on renal tissue WNTs ligands, including Wnt3a and Wnt5a. ns>0.05, versus the control group, n=5.

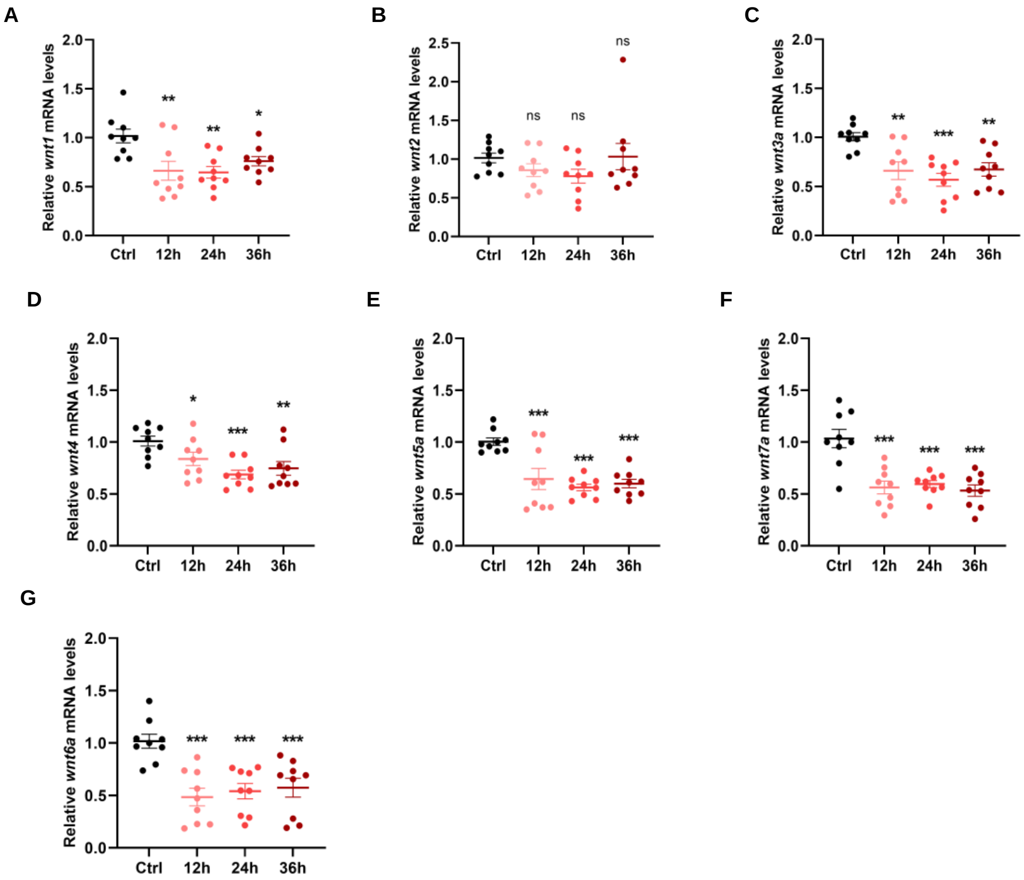

Supplementary Figure 5 Succinate reduced mRNA levels of Wnt3a and Wnt5a in the macrophage

500 μM succinate treated RAW 264.7 cells at different time points, succinate decreased mRNA levels of WNTs ligand including Wnts1, Wnt3a, Wnt4, Wnt5a, Wnt6a, and Wnt7a. ns>0.05, **P*<0.05, ***P*<0.01, ***P*<0.001, versus the control group, n=3, biologically repeated 3 times.

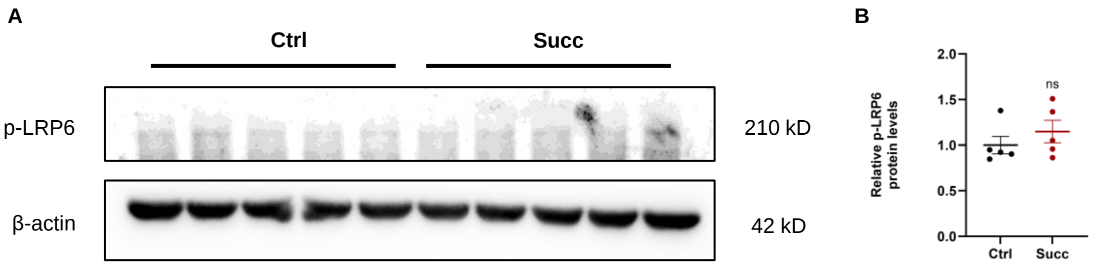

Supplementary Figure 6 Succinate had no significant stimulatory effect on renal tissue p-LRP6

The renal protein level of p-LRP6 was not altered by succinate.

ns>0.05, versus the control group, n=5.

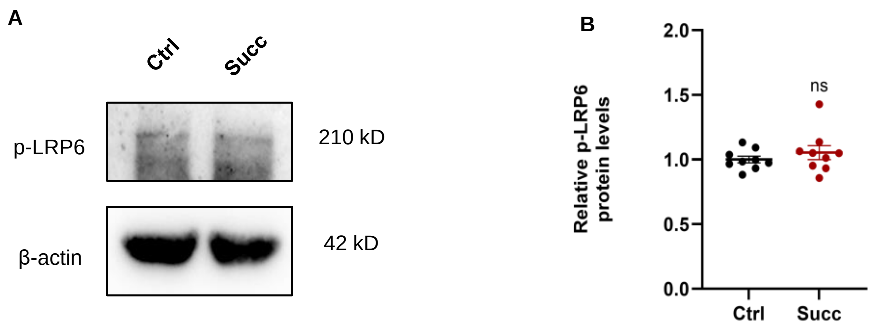

Supplementary Figure 7 Succinate had no significant stimulatory effect on renal tissue p-LRP6

500 μM succinate treated RAW 264.7 cells for 12h, and western blotting to detect the protein levels of p-LRP6. Succinate had no significant stimulatory effect on macrophage p-LRP6 protein level. ns>0.05, versus the control group, n=3, biologically repeated 3 times.

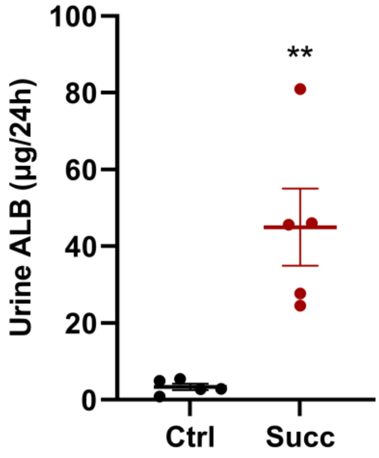

Supplementary Figure 8 Succinate caused mice proteinuria

24h urine of C57/BL 6 male mice was collected after treatment by metabolic cage and analyzed for albumin excretion. Succinate significantly promoted urine albumin excretion. ***P*<0.01, versus the control group, n=5.

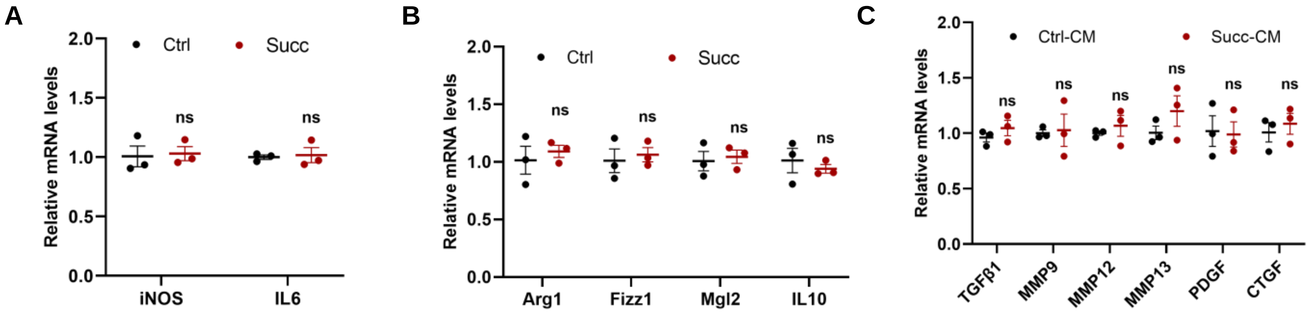

Supplementary Figure 9 Succinate did not change the mRNA expressions of macrophages-related M1,M2 markers, and profibrotic factors in HK2 cells

HK2 cells were treated with 500 μM succinate for 24h, and mRNA changes were analyzed. ns>0.05, versus the control group, n=3.

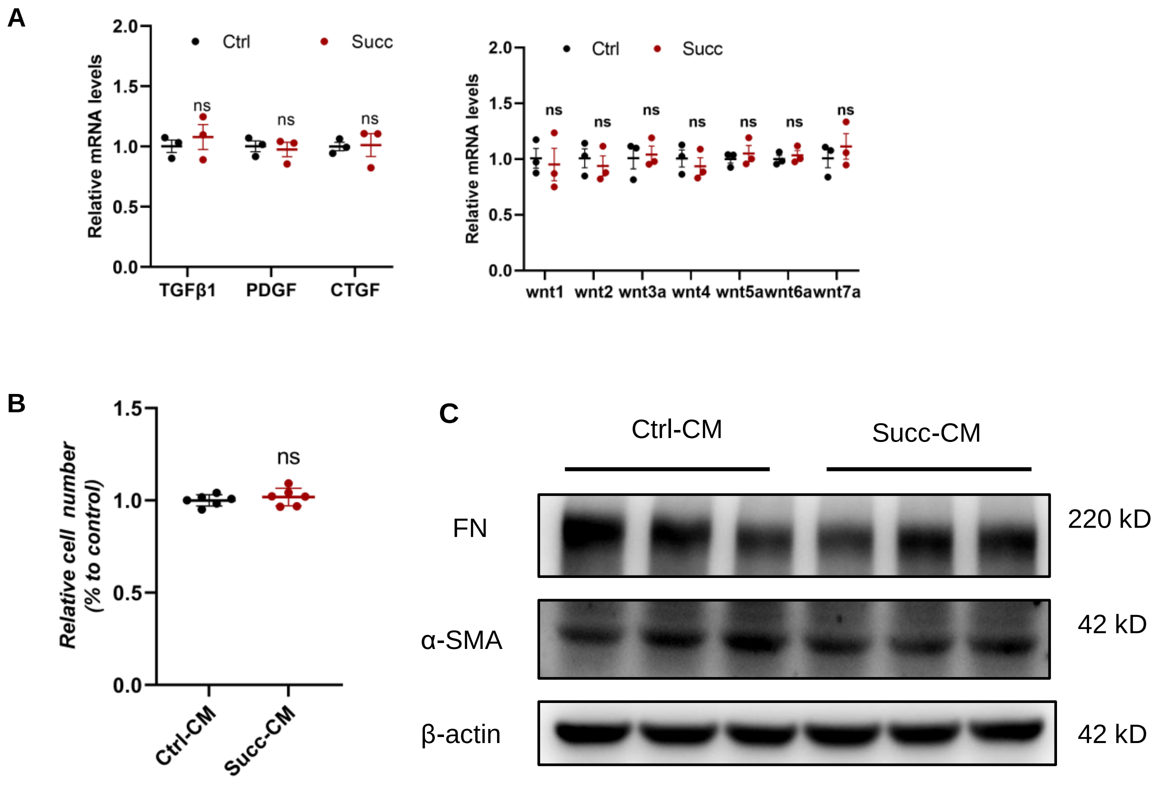

Supplementary Figure 10 Succinate treated-HK2 cells failed to enhance the proliferation and activation of NRK-49F fibroblast

(A) HK2 cells were treated with 500 μM succinate for 24h, and mRNA changes of profibrotic factors were analyzed.

(B-C) 500μM succinate was used to stimulate HK2 cells for 48h, and the conditioned medium was collected, centrifuged, and incubated with NRK-49F. The proliferation and activation of fibroblasts were detected by CCK8 assay and WB individually. ns>0.05, versus the control group, n=3.

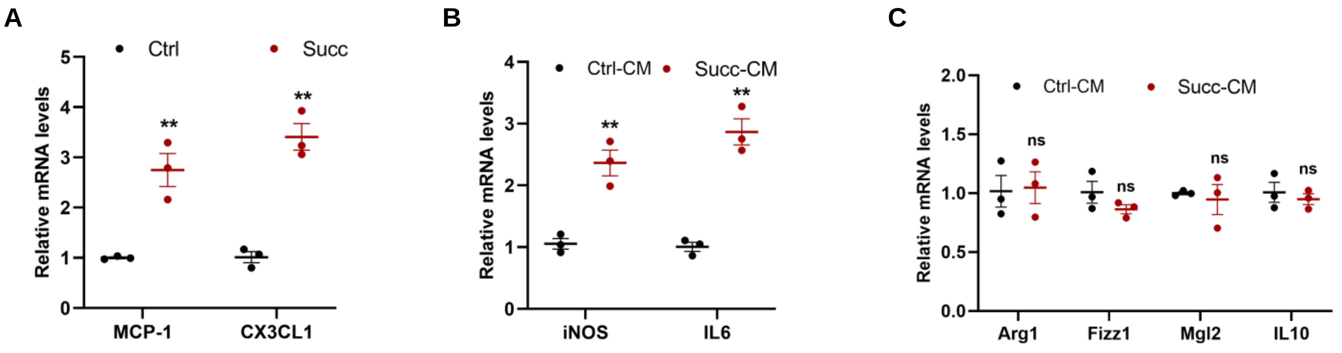

Supplementary Figure 11 Conditioned medium of HK2 cells following succinate treatment induced macrophages adopting pro-inflammatory M1 polarization

(A) HK2 cells were treated with 500 μM succinate for 24h, and mRNA changes of chemokine factors were analyzed.

(B-C) 500μM succinate was used to stimulate HK2 cells for 48h, and the conditioned medium was collected, centrifuged, and incubated with RAW 264.7 for 24h. mRNA changes of M1, M2 markers were analyzed. ns>0.05, ***P*<0.01, versus the control group, n=3.

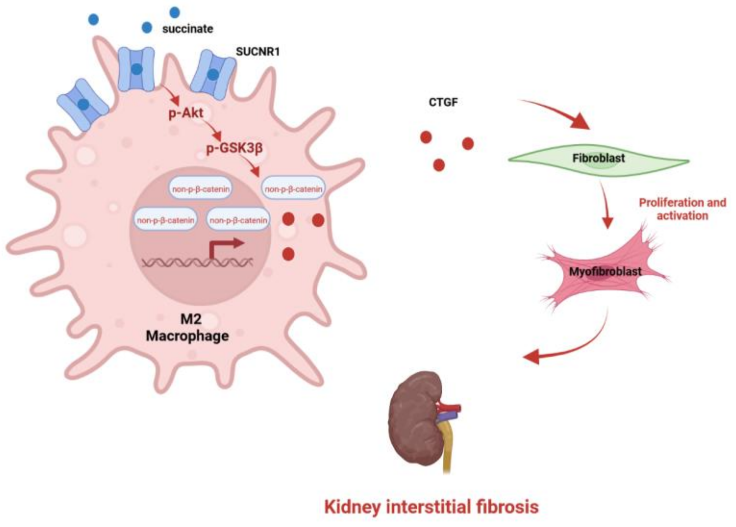

Supplementary Figure 12 The working model of succinate induces renal fibrosis

In Brief, we have shown that succinate-SUCNR1 of macrophages promoted M2 polarization and upregulation of CTGF via p-Akt/p-GSK3β/β-catenin signaling, which stimulated renal fibroblast proliferation and activation, resulting in kidney interstitial fibrosis.
