# Supplementary material for: The pathogenic role of succinate-SUCNR1: a critical function that induces renal fibrosis via M2 macrophage
Source: Cell Commun Signal. 2024 Jan 30;22:78. doi: 10.1186/s12964-024-01481-5 (PMC10826041; doi:10.1186/s12964-024-01481-5)
Supplement: Supplementary file 2 — Additional file 2. [file 12964_2024_1481_MOESM2_ESM.docx]

Gene Sequences（5^，^ to 3^，^）

m-*iNOS*-F TCATGACATCGACCAGAAGC

m-*iNOS*-R GGACATCAAAGGTCTCACAG

m-*IL6*-F TCTGGAGTACCATAGCTACCTGGAGT

m-*IL6-*R GGAGAGCATTGGAAATTGGGGTAGGA

m-*Arg1*-F CTCCAAGCCAAAGTCCTTAGAG

m-*Arg1*-R AGGAGCTGTCATTAGGGACATC

m-*Fizz1*-F CCTGCTGGGATGACTGCTA

m-*Fizz1*-R TGGGTTCTCCACCTCTTCAT

m-*Mgl2*-F TTAGCCAATGTGCTTAGCTGG

m-*Mgl2*-R GGCCTCCAATTCTTGAAACCT

m-*IL10*-F GCTCTTACTGACTGGCATGAG

m-*IL10*-R CGCAGCTCTAGGAGCATGTG

m-*CD36*-F CCTTGGCAACCAACCACAAA

m-*CD36*-R ATCCACCAGTTGCTCCACAC

m-*galectin3*-F GCCCTTGCCTGGAGGAGTCATG

m-*galectin3*-R CATTGAAGCGGGGGTTAAAGTGG

m-*PDGF*-F CGCCTGCAAGTGTGAGACAG

m-*PDGF*-R GAATGGTCACCCGAGCTTGA

m-*MMP9*-F CCTACTGCGGGCTCTTCTGA

m-*MMP9*-R CATCCACATTGCAAGGATTGTC

m-*MMP12*-F GGGCTGCAGCATTCCAATAA

m-*MMP12*-R GTCATCAGCAGAGAGGCGAAA

m-*MMP13*-F GAAGACCCCAACCCTAAGCAT

m-*MMP13*-R CGGAGACTGGTAATGGCATCA

m-*CTGF*-F GGGCCTCTTCTGCGATTTC

m-*CTGF*-R ATCCAGGCAAGTGCATTGGTA

m-*TGFβ1*-F GCGGCAGCTGTACATTGACT

m-*TGFβ1*-R GCTCCAAATATAGGGGCAGG

m-*Sucnr1*-F GGCAGAGTTTTCTGTCGAGAC

m-*Sucnr1*-R ACATTCCCAAGCAGTCCAA

m-*wnt1*-F ACTACGTTGCTACTGGCACT

m-*wnt1*-R GTTCACGATGCCCCACCATC

m-*wnt2*-F CTCTCGGTGGAATCTGGCTC

m-*wnt2-*R CCTGTAGCTCTCATGTACCACC

m-*wnt3a*-F GCTACCCGATCTGGTGGTCC

m-*wnt3a*-R CAGAGAATGGGCTGAGTGCT

m-*wnt4*-F CGAGCAATTGGCTGTACCTGG

m-*wnt4*-R CCTCAAGGTTCCGTTTGCAC

m-*wnt5a*-F CTCCGGCCCAGAAGCC

m-*wnt5a*-R TTGGAAGACATGGCACCTCC

m-*wnt6a*-F CAGGACATCCGAGAGACAGC

m-*wnt6a*-R CCCATGGAACAGGCTTGAGT

m-*wnt7a*-F GGAGCTCAAAGTGGGGAGTC

m-*wnt7a*-R CCTCCAGGATCTTGCTTCTCC
